# Towards equitable representation in long-term residential care: widening the circle to ensure “essential voices” in research teams

**DOI:** 10.1186/s40900-024-00562-6

**Published:** 2024-03-25

**Authors:** Mary Jean Hande, Prince Owusu, Katie Aubrecht, Denise Cloutier, Carole Estabrooks, Janice Keefe

**Affiliations:** 1 Trent University, Peterborough, Canada; 2 Carleton University, Ottawa, Canada; 3 St. Francis Xavier University, Antigonish, Canada; 4 University of Victoria, Victoria, Canada; 5 University of Alberta, Edmonton, Canada; 6 Mount Saint Vincent University, Halifax, Canada

**Keywords:** Long-term residential care, COVID-19, Equity, Representation, Quality of life, Lived experience

## Abstract

The COVID-19 pandemic exposed long-standing inequities in Canada’s long-term residential care (LTRC) sector with life-threatening consequences. People from marginalized groups are overrepresented among those who live in, and work in LTRC facilities, yet their voices are generally silenced in LTRC research. Concerns about these silenced voices have sparked debate around ways to change LTRC policy to better address long-standing inequities and enhance the conditions that foster dignity for those who live and work in LTRC. Weaving an analysis of historical and cultural attitudes about LTRC, and promising strategies for engaging people with lived experience, we argue that the voices of people with lived experience of life and work (paid and unpaid) in LTRC are essential for ethically and effectively shifting long-standing inequities. Lessons from a 4-year, national, multi-disciplinary research study, known as the Seniors Adding Life to Years (SALTY) project, suggest that resident-determined quality of life can be prioritized by centring the perspectives of residents, their family/friends, direct care workers, volunteers, and people living with dementia in the research process. Accordingly, we highlight strategies to include these voices so that meaningful and impactful system change can be realized.

## Plain Language Summary

This article argues that long-term residential care facilities in Canada have a long history of perpetuating social inequalities, beginning with seventeenth century poor houses and almshouses, from which long-term residential care facilities evolved in North America. We highlight that those who currently reside in long-term residential care are more likely to be people with less social power—for example women, people living with dementia, and people with low-income. These residents are rarely included in research projects as co-designers of research, co-producers of knowledge, or experts on the realities of long-term residential care. We explore strategies for addressing these underrepresented voices and inequalities in research by highlighting promising examples of resident, family, and worker-engagement emerging from a pre-pandemic to early pandemic pan-Canadian research project on quality of life in long-term residential care. We argue that long-term residential care residents, their family/friend caregivers, people living with dementia, and direct care workers have voices that are essential in residential care design and research engagement strategies. By prioritizing these voices in research, we can better amplify their critical perspectives in broader policy and decision-making processes that guide meaningful and impactful system change.

## Introduction

The pandemic devastated Canada’s long-term residential care (LTRC) sector, where more than 50% of Canada’s COVID deaths occurred [1]. Another major consequence of the pandemic was the exodus of LTRC workers in significant numbers [2]. These outcomes reflect deep fissures and pre-existing challenges arising from longstanding funding shortfalls, trends towards privatization, rigidly hierarchical organization, staff burnout, and staff shortages [3, 4]. LTRC experts, advocacy groups, and unions have been sounding the alarm for decades [5]. Yet, despite media accounts of high death tolls, prolonged staffing crises, and deplorable living conditions in some LTRC facilitities throughout the pandemic, urgently needed changes in LTRC, remain outstanding. This failure to act has amplified the distress experienced by LTRC residents, families, and direct care workers [4].

The disproportionate impact of the pandemic on those living and working in LTRC in Canada and other jurisdictions is well documented, highlighting the vulnerability of those living and working in LTRC in Canada [3, 4, 6]. Residents from marginalized groups such as those with low income, female, living with dementia, and those with complex and chronic health challenges, are *over*represented in LTRC [7, 8]. Staff who provide direct care in LTRC are also largely women, who are themselves growing older, and a rapidly growing proportion are racialized immigrant workers [9]. Despite their overrepresentation, however, the perspectives of these equity-seeking groups are generally and profoundly *under*represented on LTRC research teams and in policies and processes. In this research, we emphasize the need to include these groups with lived experience, because their voices are essential in research teams (e.g., as collaborators, members of steering or advisory committees, etc.), hence we refer to these perspectives as “essential voices”. This position towards including groups with lived experience in research teams is growing in popularity and importance [10–13].

Our literature search reveals that LTRC residents, in particular, are rarely invited to contribute as team members, advisors or research partners—although there are some notable exceptions [14]. Several recent studies [15–17] reveal that mainstream Canadian news and policy analyses of COVID-19 and LTRC seldom reflect the first-hand experiences of direct care staff and family members; LTRC resident voices and perspectives are almost entirely absent. Conversely, voices that *are* heard speaking about the challenges of LTRC, are largely politicians and clinicians, regardless of their lived experience with LTRC. The perspectives of these politicians, clinicians, and researchers carry weight as they speak to the media, determine public health measures, and guide decision-making around how, (or how not), to address the current and ongoing crisis unfolding in LTRC [15–17].

The aim of this paper is to propose alternative methods for including essential voices in LTRC research, to clarify and move beyond the inequity of underrepresentation and voice in LTRC in Canada. To build our case, we review both long-standing inequities in LTRC and highlight promising approaches in LTRC research that have the potential to amplify essential voices drawing on a case of research undertaken pre-pandemic to early pandemic, with a multi-disciplinary, multi-method, pan-Canadian research project called Seniors Adding Life to Years (SALTY), which focused on enhancing equity and quality of life in LTRC. In particular, we examine SALTY’s advisory group of essential voices—including two people living with dementia, one resident, one family caregiver, one direct care worker, and two volunteer perspectives—to highlight the ways in which SALTY facilitated conditions and relations that amplied their voices and their potential impact on the direction of LTRC research both within and beyond SALTY. Focus on the Canadian context is important given its unique regulatory frameworks, funding structures, and regional differences [18], which can make international comparisons challenging. We conclude with recommendations for meaningfully engaging essential voices in future LTRC research and, by extension, decision and policy-making processes.

## A history of inequity in LTRC

Facility-based LTRC in Canada evolved from seventeenth century almshouses and Elizabethan poor law to become a dominant model of caring for older people in North America. In those early days, the distinctions between almshouses, prisons, and poorhouses were blurred, and institutions were often intentionally inhospitable to discourage the number of people seeking and residing in such places, and the length of their stay [19, 20]. By the late nineteenth century, more than 70% of poorhouse occupants in Ontario, Canada were over 60 years old, unable to work, find housing, or without family that could provide care for them at home [21]. As the Canadian population began to grow older in the twentieth and twenty-first centuries in the context of post-World War II affluence, there was a renewed interest in the importance of social safety nets [20]. Public perceptions and discourse around these institutions began to shift towards the creation of “old-age homes,” which were portrayed as “homelike” and even “luxurious” in some cases [20], p291]. However, in reality these old-age homes were somewhere between hospitals and poorhouses [20], p284] Historian Jim Struthers [20] argued that many of the improvements made to LTRC facilities over this time period were largely cosmetic.(p287) For example, while efforts were made to market LTRC facilitaties as attractive, homelike environments, the control that LTRC residents had over their day-to-day routines and organization of the facilities remained very limited [20]. Examples of promising practices for enhancing quality of work and quality of care in Canadian LTRC systems today, include developing innovative management styles, encouraging relational care, and curbing privatization [18]. Nevertheless, the historical legacy of institutionalism, neglect, and inequity is still reflected in LTRC infrastructures, designs, policies, and delivery [21]. This is because, LTRC facilities are most often managed and operated as hospital-like institutions, rather than communities of people who need support. With this in mind, it is not surprising that the stigma and public fear of LTRC as a place of last resort, (and old age in general), has lingered [20, 22].

Over the last 50 years, efforts to improve LTRC have aimed at designing smaller scale, more homelike environments, and enhancing resident dining experiences with more flexible, nutritious, and varied meals [18]. Such efforts are often undermined by austerity measures and trends towards privatization, which have exacerbated staff shortages, and left the sector woefully underfunded, and largely neglected in policy reforms [23]. Alongside underfunding and neglect are strong societal undercurrents of discrimination, such as ageism and ableism, towards people living with chronic illness and impairment, that reinforce notions that people with health and mobility challenges do not deserve better [24–26]. Similarly, overrepresentation of low-income women and racialized workers in LTRC is also linked to sexist and racist abuse and harassment in LTRC [9, 27], which interlocks with and reinforces ageism and ableism [25]. Moreover, where LTRC is organized through top-down governance and restrictive regulations, these structural issues reduce family, staff, and volunteers’ capacities to engage in resident-centred care and enhance their quality of life [28–32]. This top-down organization also contributes to systemic inequities that reduce people to objects or purveyors of care, and justify racial and gendered inequalities and inequitable treatment of people chonric health issues [9, 23, 25], who are overrepresented in LTRC. Given this historical and cultural antecedent, it is perhaps unsurprising that women and other historically marginalized groups, particularly those with low income, remain proportionally overrepresented in LTRC facilities [7] and continue to have limited influence in terms of LTRC decision-making and policy.

COVID-19 catalyzed public interest on what needs to change in LTRC. Statistics Canada reports have captured the profound impacts of COVID-19 on LTRC in terms of staffing levels and burnout, resident acuity and death rates, ongoing infection control, and many other issues [2]. Together, recommendations from multiple provincial and federal reports and expert commentaries have been published to guide LTRC during the COVID-19 pandemic and beyond [33–37]. But despite increased attention, limited system change occurred [4]. Clearly, external pressures and action are needed. Some of this momentum can be achieved by prioritizing the perspectives and engagement of people living and working in LTRC in research, in order to shift the status quo.

## Designing LTRC research to engage and ensure essential voices are heard

In the last 15 years, LTRC researchers have started to shift research engagement strategies by using frameworks and methods that purposefully centre the perspectives and preferences of people with essential voices, to expose and address inequities in LTRC. Such strategies include advisory groups [12, 38, 39], relational ethnography [9, 40], participatory approaches [41], collaborative enquiry and co-design [10, 14], all of which enable researchers, clinicians, and decision-makers to amplify essential voice perspectives and ensure that residents, their family/friends, and direct care workers are an integral part of the research team. We focus here on the case of SALTY, a recent, pre-pandemic national research project, that used a variety methods to examine quality of life in LTRC from multiple perspectives.

SALTY's diverse range of qualitative and quantitative methods included: analysis of quantitative government LTRC data, Delphi panels composed of advisors with essential voices vocalizing priorities around burdensome symptoms in the last year of life [42], rapid ethnographic fieldwork involving tours of LTRC facilities [9, 40], supporting the use of resident-centric frameworks to analyze existing LTRC policy [43], and evaluating a palliative care intervention [44] to examine and conceptualize quality of life on multiple scales and across diverse domains. The emphasis on the resident-centric perspective, as situated within care relationships, contributed to a more equity-oriented, inclusive research approach. Attention towards understanding the experiences of those whose voices have been silenced for too long, is motivated by a deeply held belief that multiple perspectives and approaches to knowledge gathering are necessary to capture the diversity of experiences for those who live and work in LTRC.

Beginning with its grant writing in 2015 until it’s conclusion in 2021, SALTY committed to the protracted (and sometimes messy!) development of conditions, relations, and practices within research processes, that shifted the politics of representation in research in ways that might steer LTRC system change and enhance quality of life. One of these structural interventions was SALTY’s team-based integrated knowledge translation approach [39] which centred the advisory group of essential voices with lived experience in LTRC—residents, direct care staff, volunteers, non-residents living with dementia, and family members to incorporate non-academic stakeholders in the research process. SALTY used a partnership model, which prioritized meaningful engagement, where reciprocity and respect are honored and the impacts of the engagement and partnership are considered mutual, even if (inevitably) uneven at any given time. Indeed, the terminology of essential voices emerged in collaboration with the latter advisory group members to recognize their essential and unique lived experience of LTRC. The authors, as members of the SALTY team, draw on these advisors’ active participation and contributions in our following analysis.

**Fig. 1:**
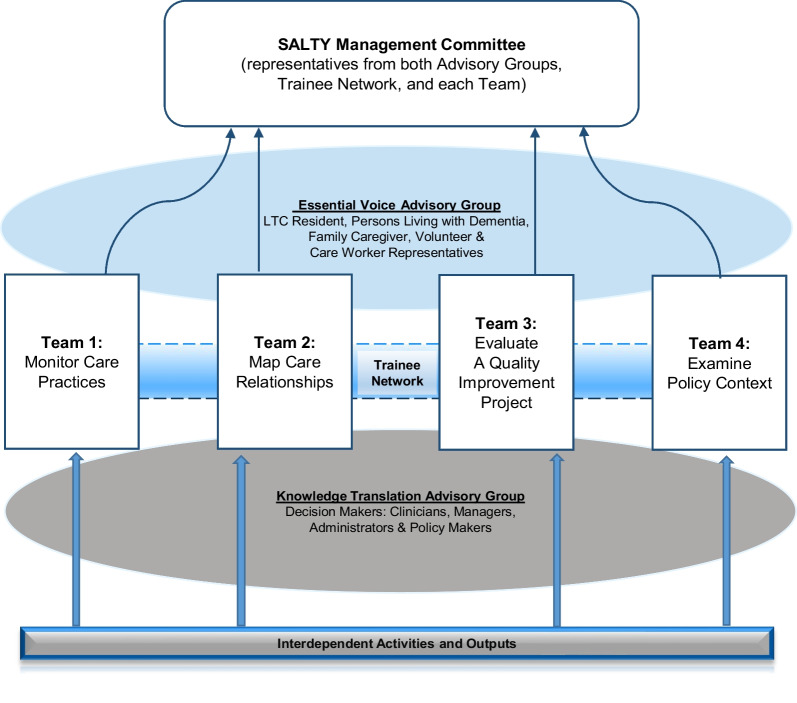
Partnership governance model

SALTY was comprised of a trainee network (including early career scholars, postdoctoral fellows, students, and research assistants), a knowledge mobilization group (composed of LTRC clinicians, managers, and policy makers), four research sub-teams spread out across four Canadian provinces, and an advisory group with representation from the groups previously named as having essential voices that must be heard, i.e. that are essential to fostering system change. These essential voices have already been noted as being *over*represented in LTRC, but historically *under*represented in research. This advisory group engaged with all sub-teams and components of the project as well as project management. (see Fig. 1).

Advisor recruitment began at the inception of the project, with six advisors (two people living with dementia, one family caregiver, one volunteer coordinator, and two direct care workers) participating in the early stages of project planning, research design, who were named as collaborators in the funding application. The compositon of the advisory group changed in the 18 months following funding approval. The volunteer coordinator was replaced because of a retirement and this new member recommended and helped recruit a resident member. Around the same time, one of the direct care workers resigned due to family responsibilities and an additional position was developed for a volunteer. Many of the essential voice advisors were recruited because of their pre-existing relationships with SALTY researchers and knowledge users, as well as various levels of experience with academic research. The majority of the advisors were based on the East Coast of Canada, where the project was based, but two members—one person with dementia and one direct care worker—lived on the West Coast. Advisors were not recruited based on gender, sexuality, or race identities, but rather their role and lived experience within the LTRC system. Nevertheless, of the final group of seven advisors, two identified as men and five as women; six identified as white and one racialized. These advisors were not asked to disclose their sexual identity or socio-economic status. Direct payment to advisors was not included in the research proposal to the funder, thus financial compensation was not provided for meeting attendance. However, SALTY organized three in-person meetings where advisors’ travel, food, and accommodation was covered by research funds. SALTY’s essential voice advisors are acknowledged in SALTY publications where they played a direct advisory role, with some publications specifically discussing the role that essential voice advisors played in analysis and knowledge production [31, 43] Advisors were co-leads in knowledge translation activities, including academic workshops [45, 46] and other project related presentations as the project wrapped up.

**Fig. 2:**
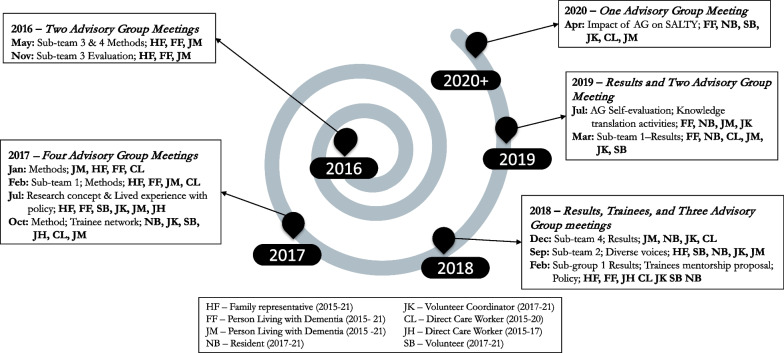
Timeline of advisory group meetings

**Fig. 3:**
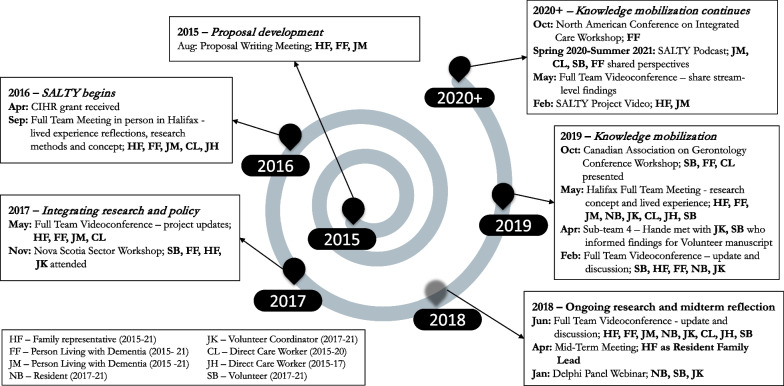
Timeline of additional advisory group activities

Our governance structure facilitated essential voice advisor representation in project decision-making as well as frequent knowledge sharing opportunities across research sub-teams and advisory groups. The advisory group met quarterly for a total of 12 group-specific meetings. These meetings often included discussions with SALTY sub-teams about various aspects of the project. Members of advisory group also participated in virtual team meetings every 6 months and two in-person meetings. Additionally, the advisory group’s chair represented the group at more than 15 project “management committee” meetings where decisions were made about the overall project organization and planning. Outside these meetings, the group engaged in a Delphi panel with one sub-team that enabled them to rank burdensome symptoms and potentially inappropriate care practices according to priority [42]. In the last advisory group meeting, we documented and shared the perceived impact of this engagement, and we continue to reflect on this in project-related writings, which are still underway. In the following section, we share key moments in the research process where the insights of the essential voices advisory group shifted the culture, conditions, and processes of the project as a whole, and prompted researchers to reflect on project priorities [see Figs. 2 and 3].

## Voice and potential impact

The impact of involving essential voice advisors in research processes is often hard to measure and validate because it requires rigorous methods for documentation and measurement [47, 48]. Unfortuantely, such measurements were not built into the SALTY process. Nevertheless, through our 5-year, expansive project, we reflected on the ways in which SALTY fostered the conditions and practices of essential voice engagement, in ways that engendered a multiplicity of perspectives, crafted new politics of representation in LTRC research, and enabled SALTY's essential voice advisors to use their “voices” to influence the direction and outcomes of SALTY’s research. All of which are important conditions for impact. Two examples highlight the importance of protracted relationship-building for meaningfully engaging essential voices in research teams. As mentioned earlier, a resident representative was not included in the advisory group until 18 monthis into the project. The inclusion of resident advisors was always a priority for the SALTY project, however the SALTY initial team members struggled to identify someone who would have the interest and required supports to leave the facility to attend SALTY meetings. When a LTRC volunteer coordinator replaced a previous member (described earlier), she soon recommended a resident with whom she had a good relationship and knew to have a keen interest in research and representation. The volunteer coordinator enabled the resident’s participation throughout the project, coordinating accessible transportation to meetings and helping to set up video calls, when meetings moved online in the early days of the pandemic. The resident advisor, who had more experience with research than the volunteer coordinator, reciprocated by supporting the coordinator to navigate the research environment and they often made joint contributions to the project. As researchers, we have learned from these advisors’ strategies to support other resident advisors with complex health and mobility challenges to actively participate in future research. We touch on these contributions in our “Implications and Recommendations” section below.

Secondly, various advisors also made targeted contributions to the SALTY project, through formal structures such as the aforementioned Delphi panel [42] and other activites, such as being consulted about volunteer roles in LTRC for an academic publication [31], and being featured in the SALTY podcast mini series “Let’s Talk Care: Fresh Perspectives on Long Term Care,” [49] led by emerging scholars who organized episodes around the the particular interests and lived experiences of the advisors. This latter project, in particular, fostered mentorship roles for essential voice advisors vis-à-vis SALTY’s Trainee Network, and deepened relationships between these advisors and emerging LTRC researchers that extended beyond the project proper. Such activities, which intentionally centred the lived experiences of our advisors, strengthened relationships and built trust within and across the SALTY team, slowly shifting normative research structures towards collaboration and mutually impactful learning and co-creation opportunities.

Engaging lived experiences in every stage of the research process and across our multiple research methodologies, sub-teams, and groups, also provided new insights into LTRC and opportunities to tip the scales on inequitable representation within LTRC knowledge- and policy-making processes. As researchers we better understand the value of active engagement and authentic listening to the essential voices of these advisors. In reflecting on the advisors' contribution to this research, we encourage researchers, both within LTRC and beyond, towards more critical forms of engagements with people living with dementia in the community, residents, and non-academics generally. SALTY’s partnership model created space for the leadership of those most impacted (and overrepresented) in LTRC to challenge common research biases and assumptions about the changes needed, and the pathways to reorganize policy-making priorities in ways that directly impact the quality of life of residents—emphasizing preferences, experiences, and accounts that would have otherwise been overlooked.

## Implications and recommendations

The SALTY project was in its last year when the COVID-19 pandemic started. As we analyzed SALTY data and reflected on our research processes, we often noted how the pandemic cyrstalized and intensified many of the equity issues we identified in our 5 years of research and team building. In particular, the need to centre resident quality of life in LTRC care research has never been more urgent and necessary, and this is difficult to do without meaningfully including essential voices in LTRC research teams. SALTY’s multi-method, multi-vocal partnership model centred a constellation of essential voices, each linked to LTRC residents, in advisory roles. This intervention revealed and underscored how resident quality of life is interrelated with quality of care, quality of work, and quality of dying and we were able to translate this into a variety of COVID reports and statements during the pandemic [37, 50–53]. SALTY also fostered new relations, conditions, practices in LTRC that ripple out into future research initiatives.

Nevertheless, we acknowledge limitations in how we documented and analyzed the impact that this essential voice engagement had in our research. The question of impact continually arose in SALTY meetings and more informal conversations with essential voice advisors as they sought clarity around their impact so they could appreciate their roles in the research and avoid essentialized and inauthentic modes of engagements. At times we struggled to recognize each advisors’ complex, overlapping, and varied roles and experiences within LTRC systems. For example, we needed to continually remind each other that each advisor had much more to contribute than simply “the volunteer perspective” or the "resident perspective" *per* *se*. Additionally, advisors did not necessarily represent the average "essential voice" either—for example our resident advisor was highly educated, did not have a dementia diagnosis, and so she shared few characteristics with the majority of residents in LTRC. As such, engagement activities needed to be reflexively tailored to fully honour those perspectives and skills present on the SALTY advisor group, while recognizing the absence of other essential voice that were not represented. As the project drew to a close, advisors reminded us in several meetings of the importance of relation-building, mentorship, and joint training for both academic researchers and essential voice adivsors to fully draw on the diverse experiences of advisors within the research process [54]. Such training would involve understanding unconscious bias, challenging and modifying normative research processes, and creating relationship-building opportunities for knowledge users, essential voice advisors, and academic researchers (see Miah et al., 2020 [55], for an example of this kind of advisor “research awareness training”).

In-person meetings and activities were preferred by most advisors because they enabled relationship-building and informal discussions. However, our resident advisor also stressed the importance of prioritizing virtual or hybrid participation for LTRC residents given the sometimes deadly consequences of exposure to infectious disease for many people with complex and chronic health challenges. She explained, safe and accessible modes of engagement, for residents in particular, must be prioritized post pandemic. This requires having relationships with staff, family, and volunteers inside LTRC facilities to help coordinate their engagement in research activities.

Finally, given the incredible diversity of LTRC residents, family, staff, volunteers, and people living with dementia, intersectionality approaches are critical to ensure essential voice advisors are meaningfully represented so that more nuanced conversations and analyses around equity and diversity in LTRC are possible. In future, we need to both build on but also look outside of existing networks when recruiting advisors to better include marginalized perspectives. Supporting these marginalized perspectives may also require further modifying normative research processes towards reflexive practices and relations attentive to race, sexuality, and embodiment, and finding ways to work productively with the inevitable tensions and discomfort of challenging dominant research practices and cultures. This also underscores the previously mentioned importance of fostering strong, lasting relations that enable essential voice advisors to represent their whole, dynamic selves, and lived experience when invited to play advisory roles in future LTRC research projects.

This research underscored that residents, family caregivers, volunteers, direct care workers, and people living with dementia need to be *meaningfully* engaged in research about LTRC as they represent essential voices that must be heard. SALTY’s methods and approaches, outlined above, provide promising examples of how this might be done in LTRC research, and similar models are emerging in other areas of LTRC research, where people with lived experience are also being engaged in the research process as advisors and knowledge developers in projects using an integrated Knowledge Translation approach (see, for example, Aubrecht et al*.* [2021] [14] and Chamberlain et al*.* [2020] [38]). Such inclusion allows essential voice advisors to engage directly with clinicians and decision-makers as equals in generating relevant and actionable knowledge to improve LTRC systems in ways that serve those most impacted by system change. Clinicians and decision-makers, in turn, stand to benefit from this direct engagement, particularly when making key decisions about LTRC improvements in resident-centred care. Bringing historically underrepresented perspectives and voices into research processes also works towards dismantling the entrenched ableism, ageism, racism, and sexism in LTRC, we discussed above.

Research funding bodies and academic institutions can play key roles in supporting this more inclusive engagement of perspectives, through targeted funding opportunities that support essential voice participation by providing honoraria, training, and accommodations, as well as training for researchers, clinicians, and decision-makers on effective engagement and relationship-building strategies. While we prioritize financial compensation for essential voice advisors in our current research, in 2014, when the SALTY research proposal and budget were drafted, such financial compensation for our valuable advisors was not considered as an expense. We agree with the Strategy for Patient-Oriented Research (SPOR), developed by the Canadian Institutes of Health Research, that engagement with advisors needs to be recognized in ways that is appropriate to their contributions [56]. The third guiding principle of the SPOR framework states “Adequate support and flexibility are provided to patient participants to ensure that they can contribute fully to discussions and decisions. This implies creating safe environments that promote honest interactions, cultural competence, training and education. Support also implies financial compensation for their involvement.” [57] Fulsome compensation (including monetary honoraria) must be provided in any project involving essential voice advisors.

While SALTY focused mostly on engaging essential voices in *research* processes, such engagement can be enhanced in LTRC policy- and decision-making as well. Currently, family and resident council mandates in some provinces have had mixed success in amplifying essential voices in LTRC to effect policy or system change. Recent research on LTRC family caregivers suggests moving beyond the council model towards more direct forms of engagement, such as integrating essential voices into LTRC decision-making processes so that they can meaningfully influence the design and operation of LTRC services and systems [58]. Similarly, we recommend more varied and direct forms of engagement for essential voices in the decision-making and management of LTRC. Such measures help shift power dynamics, democratize critical decision-making, and ensure that LTRC policies and programs attend to the needs and preferences of those most impacted by LTRC—residents, direct care staff, and resident families.

## Conclusion

LTRC’s longstanding underfunding, staff shortages, and rigid hierarchies wreaked a deadly toll during the COVID-19 pandemic, particularly for LTRC residents with complex health challenges. These structural challenges amplified intersecting arcs of societal ableism, ageism, racism, and sexism towards people living and working in LTRC. They help explain inequitable relationships between who lives, visits, works and volunteers on one hand, and who is guiding policy and shaping decision-making and knowledge production in the LTRC sector, on the other hand. Moving towards equity in LTRC requires a seismic shift in the way we structure research to engage a broad range of essential voices that are heard and prioritized in research and decision-making. While impact is often hard to measure [47], research approaches and methods that centre residents and those who care for them directly, as highlighted in our pan-Canadian SALTY study, give us concrete starting points towards a more equitable future for LTRC—a future that truly values essential voices, their relationships and life trajectories around work, care, living and dying in LTRC.

## Data Availability

The datasets used and/or analyzed during the current study are available from the corresponding author on reasonable request.

## Abbreviations

LTRC: Long-term residential care
SALTY: Seniors-Adding Life to Years
